# Supplementary material for: End Sequence Analysis Toolkit (ESAT) expands the extractable information from single-cell RNA-seq data
Source: Genome Res. 2016 Oct;26(10):1397–410. doi: 10.1101/gr.207902.116 (PMC5052061; doi:10.1101/gr.207902.116)
Supplement: Supplemental Material [file supp_gr.207902.116_Supp_Materials.tar.gz › ESAT-esat_v0.1/doc/html/classjaligner_1_1_alignment.html]

ESAT: jaligner.Alignment Class Reference


ESAT


- Main Page
- Related Pages
- Packages
- Classes
- Files

- Class List
- Class Index
- Class Hierarchy
- Class Members

All Classes Namespaces Files Functions Variables Enumerator Pages

- jaligner
- Alignment

Public Member Functions |
Static Public Member Functions |
Static Public Attributes |
List of all members

jaligner.Alignment Class Reference

|  |  |
| --- | --- |
| Public Member Functions | |
|  | Alignment () |
|  | |
| float | getExtend () |
|  | |
| void | setExtend (float extend) |
|  | |
| Matrix | getMatrix () |
|  | |
| void | setMatrix (Matrix matrix) |
|  | |
| String | getName1 () |
|  | |
| void | setName1 (String name1) |
|  | |
| String | getName2 () |
|  | |
| void | setName2 (String name2) |
|  | |
| float | getOpen () |
|  | |
| void | setOpen (float open) |
|  | |
| float | getScore () |
|  | |
| void | setScore (float score) |
|  | |
| int | getLength () |
|  | |
| char[] | getSequence1 () |
|  | |
| void | setSequence1 (char[] sequence1) |
|  | |
| char[] | getSequence2 () |
|  | |
| void | setSequence2 (char[] sequence2) |
|  | |
| int | getStart1 () |
|  | |
| void | setStart1 (int start1) |
|  | |
| int | getStart2 () |
|  | |
| void | setStart2 (int start2) |
|  | |
| int | getGaps () |
|  | |
| void | setGaps (int gaps) |
|  | |
| int | getIdentity () |
|  | |
| float | getPercentIdentity () |
|  | |
| void | setIdentity (int identity) |
|  | |
| int | getLongestPerfectMatch () |
|  | |
| int | getLongestUngappedAlignment (int maxMismatches) |
|  | |
| char[] | getMarkupLine () |
|  | |
| void | setMarkupLine (char[] markupLine) |
|  | |
| int | getSimilarity () |
|  | |
| void | setSimilarity (int similarity) |
|  | |
| String | getSummary () |
|  | |
| String | toString () |
|  | |
| float | calculateScore () |
|  | |
| int | getNumberOfMatches () |
|  | |
| int | getNumberOfMatches2 () |
|  | |
| float | getScoreWithNoTerminalGaps () |
|  | |
| boolean | checkScore () |
|  | |
| Sequence | getOriginalSequence1 () |
|  | |
| void | setOriginalSequence1 (Sequence originalSequence1) |
|  | |
| Sequence | getOriginalSequence2 () |
|  | |
| void | setOriginalSequence2 (Sequence originalSequence2) |
|  | |
| int | getGaps1 () |
|  | |
| int | getGaps2 () |
|  | |

|  |  |
| --- | --- |
| Static Public Member Functions | |
| static Alignment | add (Alignment a1, Alignment a2) |
|  | |
| static Alignment | copy (Alignment alignment) |
|  | |

|  |  |
| --- | --- |
| Static Public Attributes | |
| static final char | GAP = '-' |
|  | |

## Detailed Description

Holds the output of a pairwise sequences alignment.

Author
:   Ahmed Moustafa (ahmed.nosp@m.@use.nosp@m.rs.sf.nosp@m..net)

## Constructor & Destructor Documentation

|  |  |  |  |  |
| --- | --- | --- | --- | --- |
| jaligner.Alignment.Alignment | ( |  | ) |  |

Constructor for Alignment

Here is the caller graph for this function:

## Member Function Documentation

|  |  |  |  |
| --- | --- | --- | --- |
| static Alignment jaligner.Alignment.add | ( | Alignment | *a1*, |
|  |  | Alignment | *a2* |
|  | ) |  |  |

static

Adds this alignment to another alignment, the order is important.

Parameters
:   |  |  |
    | --- | --- |
    | a1 | The 1st alignment |
    | a2 | The 2nd alignment |

Returns
:   the sum of two alignment

Here is the call graph for this function:

|  |  |  |  |  |
| --- | --- | --- | --- | --- |
| float jaligner.Alignment.calculateScore | ( |  | ) |  |

Calculate the score of the alignment, not using the score field (the function only uses sequence1, sequence2, matrix and gap penalties).

Returns
:   the calculated score (By: Bram Minnaert)

Here is the caller graph for this function:

|  |  |  |  |  |
| --- | --- | --- | --- | --- |
| boolean jaligner.Alignment.checkScore | ( |  | ) |  |

Check if the calculated score matches the field score.

Returns
:   true if equal, else false. (By: Bram Minnaert)

Here is the call graph for this function:

|  |  |  |  |  |  |
| --- | --- | --- | --- | --- | --- |
| static Alignment jaligner.Alignment.copy | ( | Alignment | *alignment* | ) |  |

static

Copies an alignment to another alignment.

Parameters
:   |  |  |
    | --- | --- |
    | alignment | Alignment |

Here is the call graph for this function:

Here is the caller graph for this function:

|  |  |  |  |  |
| --- | --- | --- | --- | --- |
| float jaligner.Alignment.getExtend | ( |  | ) |  |

Returns
:   Returns the extend.

|  |  |  |  |  |
| --- | --- | --- | --- | --- |
| int jaligner.Alignment.getGaps | ( |  | ) |  |

Returns
:   Returns the gaps.

Here is the caller graph for this function:

|  |  |  |  |  |
| --- | --- | --- | --- | --- |
| int jaligner.Alignment.getGaps1 | ( |  | ) |  |

Returns the number of gaps of the aligned sequence #1

Returns
:   the number of gaps of the aligned sequence #1

|  |  |  |  |  |
| --- | --- | --- | --- | --- |
| int jaligner.Alignment.getGaps2 | ( |  | ) |  |

Returns the number of gaps of the aligned sequence #2

Returns
:   the number of gaps of the aligned sequence #2

|  |  |  |  |  |
| --- | --- | --- | --- | --- |
| int jaligner.Alignment.getIdentity | ( |  | ) |  |

Returns
:   Returns the identity.

|  |  |  |  |  |
| --- | --- | --- | --- | --- |
| int jaligner.Alignment.getLength | ( |  | ) |  |

Returns the length of the alignment

Returns
:   Alignment length

Here is the caller graph for this function:

|  |  |  |  |  |
| --- | --- | --- | --- | --- |
| int jaligner.Alignment.getLongestPerfectMatch | ( |  | ) |  |

Returns
:   The length of the longest contiguous perfect sub-alignment

|  |  |  |  |  |  |
| --- | --- | --- | --- | --- | --- |
| int jaligner.Alignment.getLongestUngappedAlignment | ( | int | *maxMismatches* | ) |  |

Get the length of the longest ungapped sub-alignment with at most a certain number of mismatches

Parameters
:   |  |  |
    | --- | --- |
    | maxMismatches | Max mismatches |

Returns
:   Length of longest ungapped sub-alignment with at most this many mismatches

|  |  |  |  |  |
| --- | --- | --- | --- | --- |
| char [] jaligner.Alignment.getMarkupLine | ( |  | ) |  |

Returns
:   Returns the markupLine.

|  |  |  |  |  |
| --- | --- | --- | --- | --- |
| Matrix jaligner.Alignment.getMatrix | ( |  | ) |  |

Returns
:   Returns the matrix.

|  |  |  |  |  |
| --- | --- | --- | --- | --- |
| String jaligner.Alignment.getName1 | ( |  | ) |  |

Returns
:   Returns the name1.

Here is the caller graph for this function:

|  |  |  |  |  |
| --- | --- | --- | --- | --- |
| String jaligner.Alignment.getName2 | ( |  | ) |  |

Returns
:   Returns the name2.

Here is the caller graph for this function:

|  |  |  |  |  |
| --- | --- | --- | --- | --- |
| int jaligner.Alignment.getNumberOfMatches | ( |  | ) |  |

|  |  |  |  |  |
| --- | --- | --- | --- | --- |
| int jaligner.Alignment.getNumberOfMatches2 | ( |  | ) |  |

|  |  |  |  |  |
| --- | --- | --- | --- | --- |
| float jaligner.Alignment.getOpen | ( |  | ) |  |

Returns
:   Returns the open.

|  |  |  |  |  |
| --- | --- | --- | --- | --- |
| Sequence jaligner.Alignment.getOriginalSequence1 | ( |  | ) |  |

Returns original Sequence #1

Returns
:   original Sequence #1

Here is the caller graph for this function:

|  |  |  |  |  |
| --- | --- | --- | --- | --- |
| Sequence jaligner.Alignment.getOriginalSequence2 | ( |  | ) |  |

Returns original Sequence #2

Returns
:   original Sequence #2

Here is the caller graph for this function:

|  |  |  |  |  |
| --- | --- | --- | --- | --- |
| float jaligner.Alignment.getPercentIdentity | ( |  | ) |  |

Percent identity

Returns
:   Matches / length

Here is the call graph for this function:

|  |  |  |  |  |
| --- | --- | --- | --- | --- |
| float jaligner.Alignment.getScore | ( |  | ) |  |

Returns
:   Returns the score.

Here is the caller graph for this function:

|  |  |  |  |  |
| --- | --- | --- | --- | --- |
| float jaligner.Alignment.getScoreWithNoTerminalGaps | ( |  | ) |  |

Calculate the score of the alignment without the terminal gaps.

|  |  |  |  |  |
| --- | --- | --- | --- | --- |
| char [] jaligner.Alignment.getSequence1 | ( |  | ) |  |

Returns
:   Returns the sequence1.

Here is the caller graph for this function:

|  |  |  |  |  |
| --- | --- | --- | --- | --- |
| char [] jaligner.Alignment.getSequence2 | ( |  | ) |  |

Returns
:   Returns the sequence2.

Here is the caller graph for this function:

|  |  |  |  |  |
| --- | --- | --- | --- | --- |
| int jaligner.Alignment.getSimilarity | ( |  | ) |  |

Returns
:   Returns the similarity.

|  |  |  |  |  |
| --- | --- | --- | --- | --- |
| int jaligner.Alignment.getStart1 | ( |  | ) |  |

Returns
:   Returns the start1.

|  |  |  |  |  |
| --- | --- | --- | --- | --- |
| int jaligner.Alignment.getStart2 | ( |  | ) |  |

Returns
:   Returns the start2.

|  |  |  |  |  |
| --- | --- | --- | --- | --- |
| String jaligner.Alignment.getSummary | ( |  | ) |  |

Returns a summary for alignment

Returns
:   String alignment summary

Here is the call graph for this function:

|  |  |  |  |  |  |
| --- | --- | --- | --- | --- | --- |
| void jaligner.Alignment.setExtend | ( | float | *extend* | ) |  |

Parameters
:   |  |  |
    | --- | --- |
    | extend | The extend to set. |

|  |  |  |  |  |  |
| --- | --- | --- | --- | --- | --- |
| void jaligner.Alignment.setGaps | ( | int | *gaps* | ) |  |

Parameters
:   |  |  |
    | --- | --- |
    | gaps | The gaps to set. |

|  |  |  |  |  |  |
| --- | --- | --- | --- | --- | --- |
| void jaligner.Alignment.setIdentity | ( | int | *identity* | ) |  |

Parameters
:   |  |  |
    | --- | --- |
    | identity | The identity to set. |

|  |  |  |  |  |  |
| --- | --- | --- | --- | --- | --- |
| void jaligner.Alignment.setMarkupLine | ( | char[] | *markupLine* | ) |  |

Parameters
:   |  |  |
    | --- | --- |
    | markupLine | The markupLine to set. |

|  |  |  |  |  |  |
| --- | --- | --- | --- | --- | --- |
| void jaligner.Alignment.setMatrix | ( | Matrix | *matrix* | ) |  |

Parameters
:   |  |  |
    | --- | --- |
    | matrix | The matrix to set. |

|  |  |  |  |  |  |
| --- | --- | --- | --- | --- | --- |
| void jaligner.Alignment.setName1 | ( | String | *name1* | ) |  |

Parameters
:   |  |  |
    | --- | --- |
    | name1 | The name1 to set. |

|  |  |  |  |  |  |
| --- | --- | --- | --- | --- | --- |
| void jaligner.Alignment.setName2 | ( | String | *name2* | ) |  |

Parameters
:   |  |  |
    | --- | --- |
    | name2 | The name2 to set. |

|  |  |  |  |  |  |
| --- | --- | --- | --- | --- | --- |
| void jaligner.Alignment.setOpen | ( | float | *open* | ) |  |

Parameters
:   |  |  |
    | --- | --- |
    | open | The open to set. |

|  |  |  |  |  |  |
| --- | --- | --- | --- | --- | --- |
| void jaligner.Alignment.setOriginalSequence1 | ( | Sequence | *originalSequence1* | ) |  |

Parameters
:   |  |  |
    | --- | --- |
    | originalSequence1 |  |

|  |  |  |  |  |  |
| --- | --- | --- | --- | --- | --- |
| void jaligner.Alignment.setOriginalSequence2 | ( | Sequence | *originalSequence2* | ) |  |

Parameters
:   |  |  |
    | --- | --- |
    | originalSequence2 |  |

|  |  |  |  |  |  |
| --- | --- | --- | --- | --- | --- |
| void jaligner.Alignment.setScore | ( | float | *score* | ) |  |

Parameters
:   |  |  |
    | --- | --- |
    | score | The score to set. |

|  |  |  |  |  |  |
| --- | --- | --- | --- | --- | --- |
| void jaligner.Alignment.setSequence1 | ( | char[] | *sequence1* | ) |  |

Parameters
:   |  |  |
    | --- | --- |
    | sequence1 | The sequence1 to set. |

|  |  |  |  |  |  |
| --- | --- | --- | --- | --- | --- |
| void jaligner.Alignment.setSequence2 | ( | char[] | *sequence2* | ) |  |

Parameters
:   |  |  |
    | --- | --- |
    | sequence2 | The sequence2 to set. |

|  |  |  |  |  |  |
| --- | --- | --- | --- | --- | --- |
| void jaligner.Alignment.setSimilarity | ( | int | *similarity* | ) |  |

Parameters
:   |  |  |
    | --- | --- |
    | similarity | The similarity to set. |

|  |  |  |  |  |  |
| --- | --- | --- | --- | --- | --- |
| void jaligner.Alignment.setStart1 | ( | int | *start1* | ) |  |

Parameters
:   |  |  |
    | --- | --- |
    | start1 | The start1 to set. |

|  |  |  |  |  |  |
| --- | --- | --- | --- | --- | --- |
| void jaligner.Alignment.setStart2 | ( | int | *start2* | ) |  |

Parameters
:   |  |  |
    | --- | --- |
    | start2 | The start2 to set. |

|  |  |  |  |  |
| --- | --- | --- | --- | --- |
| String jaligner.Alignment.toString | ( |  | ) |  |

## Member Data Documentation

final char jaligner.Alignment.GAP = '-'

static

Gap character

---

The documentation for this class was generated from the following file:

- java/jaligner/Alignment.java


---

Generated on Fri Aug 15 2014 15:11:05 for ESAT by  

 1.8.7
